# Health comparison between guinea pigs raised in uncontrolled and controlled environments

**DOI:** 10.14202/vetworld.2022.1575-1582

**Published:** 2022-06-29

**Authors:** Laksmindra Fitria, Nastiti Wijayanti, Tuty Arisuryanti, Siti Isrina Oktavia Salasia

**Affiliations:** 1 Laboratory of Animal Physiology, Department of Tropical Biology, Faculty of Biology, Universitas Gadjah Mada, Yogyakarta, Indonesia; 2 Laboratory of Genetics and Breeding, Department of Tropical Biology, Faculty of Biology, Universitas Gadjah Mada, Yogyakarta, Indonesia; 3 Department of Clinical Pathology, Faculty of Veterinary Medicine, Universitas Gadjah Mada, Yogyakarta, Indonesia

**Keywords:** clinical biochemistry, environment, guinea pig, health status, hematology, parasites

## Abstract

**Background and Aim:**

Guinea pigs (GPs) (*Cavia porcellus*) are not only kept as pets but also widely used in biological and biomedical research. At present, GPs are also used as a species for animal-assisted therapy (AAT). Consequently, assessing their health status is vital to determining their quality of life, usability for research, and prevention of spread of potential zoonotic diseases to patients using them for AAT. GPs are mainly sourced from animal markets supplied by traditional farms, where environmental factors and sanitation are not properly controlled. This study aimed to compare health status between GPs raised in uncontrolled (conventional farm) and controlled (animal facility) environments.

**Materials and Methods:**

Sample animals were obtained from a local animal market and transported to an animal facility. After 1 week of acclimatization, the health status of the animals, including general health condition, body weight, body temperature, complete blood count, liver function (alanine aminotransferase and bilirubin), renal function (blood urea nitrogen and creatinine), and presence of ectoparasites and endoparasites, was assessed. Then, the animals were maintained in the animal facility following the standard procedure for laboratory animals. After 2 months, the animals’ health status was re-examined, assessing the same parameters.

**Results:**

Based on the evaluated parameters, GPs raised in an uncontrolled environment were found to have poorer health status than those raised in a controlled environment. There were significant differences in almost all parameters between GPs raised in controlled and uncontrolled environments. We found that the populations of two ectoparasites, *Gyropus ovalis* and *Gliricola porcelli*, and one endoparasite, *Eimeria caviae*, decreased significantly following the movement of the animals from an uncontrolled to a controlled environment.

**Conclusion:**

GPs raised in an uncontrolled environment have poor health status. However, a controlled environment with better care management can improve the health status of GPs.

## Introduction

*Cavia porcellus*, or guinea pigs (GPs), are tropical animals that originated from the Andes Mountains, South America. The species we are familiar with today was derived from wild animals domesticated sometime between 6000 and 2000 BC by Central Andeans, who raised GPs, particularly to serve as the main source of protein in their daily diet and for medicinal purposes [1]. Cavy is the proper but less popular name for GP; they are also known as Dutch rats because Dutch and Spanish traders introduced these animals to Europe, Africa, and the rest of the world, including Indonesia, in approximately 1554 [2, 3]. Outside South America, GPs are kept as exotic pets [4]. GPs have been used as experimental animals since 1780 for research on pathology, nutrition, genetics, pharmacology, allergies, radiology, immunology, and other fields [5]. Furthermore, GPs are used as animal models in dentistry, osteology, nutrition, and physiology and as models for various infectious diseases [6]. Recently, GPs have been involved in animal-assisted therapy (AAT) for physical and mental treatment, similar to cats, dogs, and horses. GPs have been used for therapeutic purposes in geriatrics-gerontology patients, individuals with chronic diseases, and children with typical development and autism spectrum disorders [7, 8].

Whether kept as pets, used for research, or used for AAT, the health status of GPs must be verified because it determines their quality of life and the validity of research data. In addition, preventing infections in owners, caretakers, and patients are also crucial because GPs are suspected of transmitting infections to humans, particularly dermatophytosis, which can pose a serious zoonotic risk to individuals who actively interact with GPs [9]. Environmental factors and husbandry/care management practices influence the physiological condition of animals and their health status [10]. Parameters that do not meet the requirements may adversely affect animal health. Unfortunately, some animals, including GPs, do not always show visible signs of illness when casually observed or clinically examined [11]. Blood tests can be used to study the physiological condition and health status of animals, including disease diagnosis, and these tests include hematology and clinical biochemistry parameters [12]. Detecting parasites in GPs will help interpret results from blood tests, both ectoparasites [13, 14] and endoparasites [15, 16].

Therefore, this study aimed to compare the health status of GPs raised in an uncontrolled environment with that of those moved to a controlled environment.

## Materials and Methods

### Ethical approval

All procedures regarding animal handling, care, and sampling methods were approved by Research Ethics Committee of Faculty of Veterinary Medicine, Gadjah Mada University at Yogyakarta, Indonesia, issued with Ethical Clearance No. 0016/EC-FKH/Eks./2020.

### Study period and location

This study was conducted from July to September 2020 at “Animal House”, the animal facility of Faculty of Biology, Gadjah Mada University (UGM), for animal maintaining, weighing, and measuring body temperature. Hematological analysis and parasite identification were performed at Laboratory of Animal Physiology, Faculty of Biology UGM. Evaluation of liver and renal function was carried out at The Integrated Research and Testing Laboratory (LPPT UGM).

### Animals

Eighteen GPs were obtained from a local animal market in Yogyakarta, Indonesia, provided by multiple vendors. The animals were kept in unstandardized animal facilities according to the standard guideline of animal welfare (uncontrolled environment). We chose individuals of American/English shorthair tricolor breed for uniformity. Based on morphological observation for sex determination, we separated into boars and sows to avoid breeding between animals. Individuals were randomly chosen with the consideration of 200–300 g for initial body weight.

### Experimental design

GPs were kept in pens based on sex; each pen consisted of three animals. One week after acclimatization, biological and health parameters were obtained from GPs raised in an uncontrolled environment. Then, GPs were moved into an animal room and kept there for 2 months. Similar biological and health parameters were obtained from GPs raised in a controlled environment.

### Procedures

Animals were transported from a local market to an animal facility at the Faculty of Biology, Gadjah Mada University, Yogyakarta, Indonesia, through land. One week after acclimatization, all animals were examined to determine their health status in an uncontrolled environment.

At the animal facility, the animals were maintained using the standard recommended procedure for husbandry/care, management, and welfare of GPs, including housing, diet, drinking water, temperature, lighting, and sanitation [17–21]. After 2 months, the animals were re-examined to determine their health status in a controlled environment.

### Biological and health parameters

The parameters used to assess the health status of animals included general health condition through physical examination, body weight, core temperature, complete blood count, liver function (i.e., alanine aminotransferase [ALT] and bilirubin levels), and renal function (i.e., creatinine and blood urea nitrogen [BUN] levels). The results were compared between the first and second assessments and evaluated using international/general [4] and local/Indonesian [17] references. Furthermore, we examined the animals for the presence of parasites. Ectoparasites were harvested from the skin, fur, and all body surfaces as per the standard parasitological method [22], whereas endoparasites were collected from fecal samples using the flotation and sedimentation methods [23].

### Blood sample collection and analysis

No more than 0.5 mL of blood was collected by clipping the toenails. Next, ethylenediaminetetraacetic acid was added as an anticoagulant for the blood samples [21, 24–26]. A complete blood count was performed using a fully automated hematology analyzer (Sysmex^®^ XP100, Jakarta, Indonesia). Bleeding and coagulation times were measured manually using a stopwatch. Then, the blood samples were centrifuged at 1400× g for 15 min in a commercial centrifuge (Eppendorf^®^5418R, Selangor, Malaysia) to yield plasma for evaluating liver and renal functions. The levels of ALT, bilirubin, creatinine, and BUN were measured using the respective kits (DiaSys^®^, Jakarta, Indonesia) and a semi-automated clinical chemistry analyzer (Microlab^®^ 300, Puteaux, France).

### Statistical analysis

Quantitative data were tabulated in a spreadsheet using Microsoft^®^Excel^®^ 2019 (Microsoft Corporation, USA) and statistically analyzed using the Statistical Package for the Social Sciences (SPSS) version 23 (IBM Corp., NY, USA), for descriptive statistics (mean ± standard deviation). Data were analyzed using a one-way analysis of variance and then Duncan’s *post hoc* test (a = 0.05) to compare results among the groups [27, 28]. Qualitative data have been reported as figures with corresponding descriptions.

## Results

### General appearance

**Figure-1:**
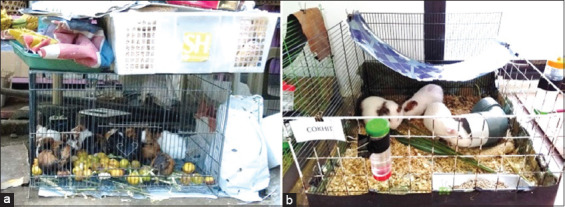
Housing of guinea pigs (GPs). (a) GPs in uncontrolled environment and (b) GPs in controlled environment.

The GPs in the market were classified as the uncontrolled environment group. The vendors used wire cages as GPs enclosures, which are commonly used to house dogs and cats. GPs were crowded inside the enclosure; the entire cage floor was occupied with no vacant spaces. When visitors approached the cage, GPs rushed to a corner and gathered together. Furthermore, boars (male GPs) and sows (female GPs) and individuals of different ages were placed inside the same cage. When the animals were palpated/examined, we felt their spines and ribs, with their hips being prominent. This result corresponds to the emaciated criteria based on GPs body condition scoring [29]. Their fur or coat looked dull and felt rough, and on some parts of the body, lesions, such as sores or scabs, were observed. Healthy GPs have clean coats because of their intense grooming behavior. In dirty environments, they groom more frequently. Grooming is also the expression of distress and response to ectoparasites, which cause itchiness on the skin [30]. The poor appearance of GPs in the markets was probably because of poor health conditions; thus, they did not properly groom. Interviews with stall owners revealed that the animals were fed only a modest diet of vegetables or fruits. Traditional GPs farmers usually do not know the impact of environmental parameters, nutrition, sanitation, and housing on the health and well-being of the animals they sell. The condition of the GPs in the uncontrolled environment group is shown in Figure-1a.

Figure-1b shows the controlled environment group, which consisted of three GPs of the same sex. The animals were placed in a pen (cage without a lid) with a size of 60 × 60 × 23 cm, which was equipped with wood shaving as bedding, a feeder, and a water bottle with a tube sipper and enriched with a tunnel, shelter, twigs or other media for gnawing, and hay or straw. These components were provided based on recommendations for standard GPs housing to meet animal welfare. Environmental parameters for the animal room, as per standard recommendations, were as follows: Room temperature, 25°C–27°C; relative humidity, 60–77%; closed circulation system with air conditioner and exhaust fan; illumination with artificial light from a 7-watt LED lamp; light intensity, 130 lux (room) and 30–60 lux (inside pen); photoperiod, 12 h dark:12 h light; and noise intensity, 30–102 dB (0.01–12.5 kHz) and 50–75 dB (12.5–70 kHz). For sanitation, we replaced the bedding twice a week. Once a week, the pens were cleaned and sanitized with detergent and disinfectant. We fed GPs with washed/rinsed cleaned fresh vegetables (i.e., cucumber, carrot, cabbage, and green leaves), grass, and hay, combined with commercial pellets. Commercial mineral drinking water was provided *ad libitum*. Furthermore, we added Vitamin C to drinking water to meet the daily ascorbic acid requirement (5–30 mg/kg/day) [31]. Similar to humans, GPs cannot synthesize endogenous Vitamin C; thus, an unbalanced diet will affect their health status (resulting in “scurvy”) and prolong the recovery process in case of an infection or disease [31, 32].

### Biological data and blood analysis

Through general health examination from physical observation and blood analysis, we compared the health status between GPs from the market and those that were moved to an animal facility (Table-1).

**Table 1:** Comparison of values of several variables for health status indicators between GP from uncontrolled environment (animal market) and after being moved to controlled environment (animal room).

Variables	Boars (n = 9)		Sows (n = 9)	
	
	Animal market	Animal room	Animal market	Animal room
BW (g)	250.00 ± 12.50^a^	446.67 ± 22.33^b^	291.67 ± 14.58^a^	436.67 ± 21.83^b^
BT (°C)	37.87 ± 1.89^b^	36.30 ± 1.82^a^	37.53 ± 1.88^a^	37.67 ± 1.88^a^
Erythrocyte profile				
RBC (×10^6^/mL)	4.48 ± 0.22^ab^	4.54 ± 0.23^ab^	4.90 ± 0.25^b^	4.28 ± 0.21^a^
HGB (g/dL)	11.72 ± 0.59^a^	12.30 ± 0.62^b^	13.12 ± 0.66^ab^	11.90 ± 0.60^a^
HCT (%)	39.08 ± 1.95^b^	37.37 ± 1.87^a^	39.62 ± 1.98^b^	36.70 ± 1.84^a^
MCV (fL)	87.23 ± 4.36^bc^	82.31 ± 4.12^ab^	80.86 ± 4.04^a^	85.75 ± 4.29^b^
MCH (pg)	26.16 ± 1.31^a^	27.09 ± 1.35^b^	26.78 ± 1.34^a^	27.80 ± 1.39^b^
MCHC (g/dL)	29.99 ± 1.50^a^	32.91 ± 1.65^b^	33.11 ± 1.66^b^	32.43 ± 1.62^b^
Leukocyte profile				
WBC (×10^3^/mL)	6.50 ± 0.33^b^	5.60 ± 0.28^a^	6.70 ± 0.34^b^	6.20 ± 0.31^ab^
NEU (×10^3^/mL)	3.60 ± 0.18^c^	2.20 ± 0.11^ab^	3.40 ± 0.17 ^c^	1.00 ± 0.05^a^
LYM (×10^3^/mL)	2.90 ± 0.15^a^	3.40 ± 0.17^b^	3.30 ± 0.17^b^	5.20 ± 0.26^c^
NEU (%)	55.38 ± 2.77^d^	39.29 ± 1.96^b^	50.75 ± 2.54^c^	16.13 ± 0.81^a^
LYM (%)	44.62 ± 2.23^a^	60.71 ± 3.04^b^	49.25 ± 2.46^ab^	83.87 ± 4.19^c^
N/L	1.24 ± 0.06^c^	0.65 ± 0.03^b^	1.03 ± 0.05^c^	0.19 ± 0.01^a^
Thrombocyte profile				
PLT (×10^3^/mL)	200.33 ± 10.02^a^	394.67 ± 19.73^b^	323.50 ± 16.18^b^	260.00 ± 13.00^a^
BT (s)	403.33 ± 20.17^b^	350.00 ± 17.50^a^	337.50 ± 16.88^a^	360.00 ± 18.00^ab^
CT (s)	140.00 ± 7.00^b^	110.00 ± 5.50^a^	127.50 ± 6.38^a^	125.00 ± 6.25^a^
Evaluation of liver functions				
ALT (U/L)	68.70 ± 3.44^b^	64.20 ± 3.21^b^	80.80 ± 4.04^c^	46.10 ± 2.31^a^
BIL (mg/dL)	1.38 ± 0.07^c^	0.63 ± 0.03^b^	0.75 ± 0.04^b^	0.36 ± 0.02^a^
Evaluation of renal functions				
CRE (mg/dL)	1.56 ± 0.08^c^	1.05 ± 0.05^b^	0.71 ± 0.04^a^	0.64 ± 0.03^a^
BUN (mg/dL)	26.22 ± 1.31^b^	14.66 ± 0.73^a^	40.14 ± 2.01^c^	11.91 ± 0.60^a^
Parasites				
*Gyropus ovalis*	+++	+	+++	+
*Gliricola porcelli*	++++	++	++++	++
*Eimeria caviae*	+++	-	+++	-

BW=Body weight, BT=Body temperature, RBC=Red blood cell, HGB=Hemoglobin, MCV=Mean corpuscular volume, MCH=Mean corpuscular hemoglobin, MCHC=Mean corpuscular hemoglobin concentration, WBC=White blood cell, NEU=Neutrophil, LYM=Lymphocyte, N/L=Neutrophil/lymphocyte ratio, PLT=Platelet, BT=Bleeding time, CT=Coagulation time, ALT=Alanine aminotransferase, BIL=Bilirubin, CRE=Creatinine, BUN=Blood urea nitrogen, The same letter following the value in a row indicates no significant difference (p > 0.05) compared to other values of the similar variable in the row

### Parasites

**Figure-2:**
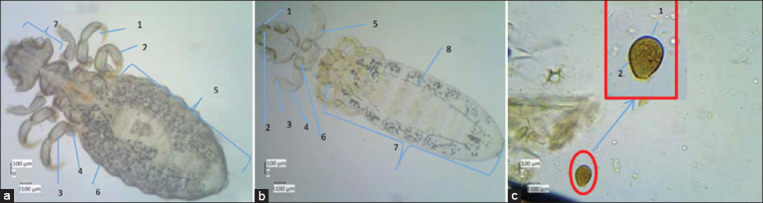
Parasites in guinea pigs (GPs) from uncontrolled environment and after moved to controlled environment. (a) *Gyropus ovalis*, (b) *Gliricola porcelli*, and (c) *Eimeria caviae*.

We examined the animals for the presence of ectoparasites and endoparasites. Ectoparasites cause skin diseases, such as scabies and infections related to environmental hygiene, such as fungal infections or dermatophytosis [13, 14]. Endoparasites mainly live in the gastrointestinal environment or bile ducts. Excessive population leads to diarrhea, bloating, and weight loss because of decreased appetite [15, 16]. We found two ectoparasites, *Gyropus ovalis* (chewing lice) and *Gliricola porcelli* (yellow lice), and one endoparasite, *Eimeria caviae* (Figure-2).

## Discussion

### General appearance

For 2 months, we raised GPs as the controlled environment group in the animal facility, following the standard guidelines for care and management that refer to their basic needs, particularly housing, environmental factors, sanitation, and welfare [17–21]. During the study, the boars and sows in the controlled environment group significantly gained weight and exhibited improvement in their appearance. At first, the GPs from the market frequently scratched their body; however, this habit decreased gradually after moving and maintaining them in a controlled environment. This activity is related to the population of ectoparasites in their body [4, 33]. Although they are easily startled and scared, GPs can adapt well when handled well. GPs are inquisitive animals by nature but dislike changes in their environment. However, they learn quickly after observing the behavior of other individuals [4, 17]. For instance, when we provided pellets, they required a few days to begin eating and enjoying them eventually. Furthermore, they required a longer period to familiarize themselves with drinking water from bottles. Feral GPs in their natural environment neither eat pellets nor drink water because they already get water from consuming vegetables or fruits.

### Housing

The habitat or environment where an animal lives, including housing and environmental parameters, predisposes its physiological condition and behavior, which determines its health status and welfare [34–36]. Transportation, transfers from one environment to another, cage structure, or housing method can trigger stress as indicated by weight loss and elevated blood cortisol levels. The population or colony size per pen also affects the welfare of GPs [36]. GP as a social animal, cannot be kept alone; at least two individuals or a group of the same sex should be housed in each pen for maintenance purposes. For breeding purposes, GPs can be caged in a pair or harem [17–20, 34]. GP is a rodent, similar to rats and mice, which keep moving about for exploration and foraging, as well as gnawing, digging, and hiding. Therefore, cages must be designed and enriched with media to facilitate these behaviors. The number of individuals in one cage must be managed, for example, avoiding overcrowding, to ensure that there is enough area for doing activities and expressing normal behavior. The pen that we modified for the controlled environment group meets the basic requirements for GPs (Figure-1b). The number of animals per pen is considered ideal for applying as a reference for keeping GPs indoor as pets or in animal facilities for research purposes [34–36], compared with the uncontrolled environment group (Figure-1a). Stress and overpopulation reduce immunity and resistance to diseases, which can be observed as elevated body temperature. Elevated core temperatures significantly indicate fever and systemic inflammation. In addition, the total and differential leukocyte counts provide data for assessing infection and immune responses [37, 38].

### Body weight and body temperature

Results showed that the body weight of the boars and sows in the controlled environment group increased significantly compared to those of the boars and sows in the uncontrolled environment group. Raised body weight indicates that animals were not stressed; they were able to express their normal behavior, including feeding well in order to grow normally. Body weight represents animal physiological status and is an important indicator for their normal growth. Naturally, animals gain weight as they age. Feed adequacy, including types of food and nutritional contents, health conditions, stress, welfare, and environmental factors, affects the feed intake and thus alters the growth rate of animals [39]. We cannot provide information on the exact age of the GPs we used in this study as the vendor did not have the birth record of their animals – a common problem in the conventional animal farming system. GPs weighing 250–350 g are approximately 2–3 months old [39–41]. GPs can be bred when their body weight reaches 400–450 g [17, 41, 42].

In the uncontrolled environment group, the boars had higher body temperature than the sows. After being moved to a controlled environment, their body temperature was significantly lower than that of the sows. Normally, male mammals have lower body temperature than females. Female sex hormones, such as estrogen and progesterone, are responsible for regulating and maintaining warm temperature in female mammals [43]. High body temperature in boars in uncontrolled environments could occur because of stress, crowdedness, and diseases. Proper care management helped regulate their body temperature back to normal ranges [36].

### Blood parameters

Blood, including both cell components and the plasma/serum, is important biological samples. Blood plays a key role in animal physiology, including the exchange of respiratory gases (oxygen and carbon dioxide), transportation of nutrients and metabolic wastes, and distribution of various endogenous products, such as enzymes, hormones, and other substances, to support an individual’s normal physiology. Circulating blood also distributes exogenous substances or xenobiotics, such as drugs, toxicants, and contaminants. Therefore, blood reflects the physiological condition and health status of animals [44, 45]. Our results demonstrated alterations in almost all blood parameters, which indicated the improvement in the health of the GPs in the controlled environment group. Values showing significant differences between the uncontrolled and controlled environment groups reassured that care and management play a major role in the health status of GPs. Hematology analysis showed some changes in the values of erythrocytes, leukocytes, and platelets. Variations in hematology and biochemical parameters are sex and age related. The values are significantly different between young and adult individuals, which can be determined as below and over 300 days [46]. Because some values in this study were significantly different and were maintained within the normal range and the growth is < 300 days, the difference reflected normal physiological dynamics.

In addition to assessing the physiological condition of animals (physical health), hematology profiles can also be used to determine their psychological condition (mental health) through indicators of stress, by calculating the ratio of neutrophils to lymphocytes (N/L). Physiological stress because of changes in environmental conditions, diseases, or other interventions will affect the psychological status of animals, manifested as distress. In general, stress in vertebrates decreases the number of lymphocytes, with a concomitant increase in the number of neutrophils; therefore, stressed animals have a high N/L ratio [47, 48]. Land transportation and the transfer of GPs from their old habitat (uncontrolled environment) to a new habitat (controlled environment), as well as changes in lifestyle and diet had the potential to cause distress [49]. Our results showed that GPs in the controlled environment group did not experience stress during the experiment, indicated by an increase in lymphocyte count and percentage, whereas neutrophil count and percentage decreased; thus, the N/L value was lower.

Liver and renal functions had decreasing values for all indicators. This is probably because cells making up both organs of GP in the controlled environment group have improved, in consequence, optimizing their normal functions. The liver is an important organ for assessing the health status of both humans and animals as it detoxifies toxic substances that enter the body and excrete waste products of the body during normal metabolism. However, these processes cause several damage to the liver structure, which can be indicated by elevating intracellular enzymes, such as ALT, because many liver cells experience necrosis. Furthermore, this injury leads to liver dysfunctions, which can be detected by elevating plasma bilirubin. The kidney is also an essential organ for determining the health status of an individual because of its functions for ultrafiltration, reabsorption, and excretion. Therefore, disruption to these processes indicates deterioration in renal function, which can be observed structurally and/or functionally. Creatinine and BUN are two main indicators for evaluating renal function [44, 50].

Published reference values for normal or healthy GPs in Indonesia are still rare. The only source was written by Smith and Mangkoewidjojo [17], which aged more than three decades ago, and does not include a wide range of parameters. International publications provide newer and more complete values and are constantly updated; however, the data were GPs that were housed in conditions that are different from husbandry and management practiced in Indonesia [4].

### Parasites

Endoparasitic infection and ectoparasitic infestation can be found in conventionally sourced and housed GPs [4, 17, 50]. In the animals we studied, we found two ectoparasites, namely, *G. ovalis* (chewing lice) and *G. porcelli* (yellow lice), and one endoparasite, namely, *E. caviae* (Figure-2). *G. ovalis* and *G. porcelli* are common ectoparasites found in GPs. *Trixacarus caviae*, *Chirodiscoides caviae*, and *Demodex caviae* are also specific ectoparasites in GPs [13, 14]; however, they were not found in the animals we studied. *E. caviae*, the main protozoa causing coccidiosis in GPs [4, 51], has been reported in Indonesia [17] and Brazil [52]. However, this parasite has not been found in African GPs; the most common infection in African GPs is caused by *Giardi*a spp. and *Cryptosporidiu*m spp. [15] and *Paraspidoder*a spp. [16].

*G. ovalis* and *G porcelli* live beneath the skin, eating epithelial cells, digging holes, and sucking blood. When consuming the epidermis, they secrete substances that induce inflammation. The itchy sensation causes GPs to scratch their skin intensively, which results in patchy hair loss, ulcerative lesions, and redness of the skin. The skin may appear dry or oily, thickened, and crusted. Severely infected animals can develop secondary infections, get stressed, and lose weight. These lice may predispose animals to health deterioration due to internal parasites, infectious diseases, poor nutrition, and poor sanitation [53]. *E. caviae* is a coccidia found specifically in GPs, with infections established through contaminated food. *Eimeria* is found in the epithelial lining or tissues in the digestive tract; sometimes, it is also found in the bile duct or renal tubules. This genus is relatively harmless; however, if the population of the organisms increases, clinical signs may occur, such as mucus in feces, diarrhea, bloody feces, colitis, anemia, and weight loss. This coccidia is not zoonotic and can be prevented with the provision of clean food and sterile bedding [17, 53]. These three parasites were found in GPs that had just arrived from the market (uncontrolled environment), during acclimatization, and also after 2 months of housing in the animal facility (controlled environment). The difference was the total number of parasites. After housing and maintenance in our controlled animal room, the population of ectoparasites dropped significantly; meanwhile, the population of endoparasite was eliminated. Sanitation plays a major role in the level of parasitic infestations and infections in GPs. Better management of animal husbandry/care and health monitoring and appropriate medical treatments by the attending veterinarians also affect the health status of animals.

*G. ovalis*, *G. porcelli*, and *E. caviae* are natural infective organisms in GPs that have occurred for a long time since domestication. As these parasites are very infectious among GP populations, good management are essential to prevent and control the diseases in GPs, including proper housing, ventilation, cleanliness, temperature, humidity, and well-balanced diets [35, 36, 53]. Multivitamins were administered to GPs that appeared weak, passive, or unhealthy. For parasites, we treated GPs with the administration of antiparasitics, such as ivermectin, combined with anti-inflammatory agents and vitamins [51, 54].

Biological data for health monitoring in GPs are already available from various countries. However, no publications have provided comprehensive data for comparing the biological and health profiles of GPs based on different environments. Our study provides new insight that differences in environmental factors and care methods greatly affect the health status of animals, which is related to sex. Our study could be used to prepare the health status of animals ready to be used as experimental animals for various research purposes.

## Conclusion

GPs raised in an uncontrolled environment (conventional farms) have poor health status according to the evaluated parameters. Moving them to a controlled environment (animal facilities) with better care management can improve their health status.

## Authors’ Contributions

SIOS and LF: Conceived and designed the study and wrote and revised the manuscript. LF: Conducted the experiments, collected samples, and analyzed the data. TA and NW: Analyzed the data. All authors have read and approved the final manuscript.
